# T cell recognition of *Mycobacterium tuberculosis* peptides presented by HLA-E derived from infected human cells

**DOI:** 10.1371/journal.pone.0188288

**Published:** 2017-11-27

**Authors:** Curtis McMurtrey, Melanie J. Harriff, Gwendolyn M. Swarbrick, Amanda Duncan, Meghan Cansler, Megan Null, Wilfried Bardet, Kenneth W. Jackson, Deborah A. Lewinsohn, William Hildebrand, David M. Lewinsohn

**Affiliations:** 1 Department of Microbiology and Immunology, University of Oklahoma Health Science Center, Oklahoma City, OK, United States of America; 2 VA Portland Health Care System, Portland, OR, United States of America; 3 Department of Pulmonary and Critical Care Medicine, Oregon Health and Sciences University, Portland, OR, United States of America; 4 Department of Pediatric Medicine, Oregon Health and Sciences University, Portland, OR, United States of America; University of Cape Town, SOUTH AFRICA

## Abstract

HLA-E is a non-conventional MHC Class I molecule that has been recently demonstrated to present pathogen-derived ligands, resulting in the TCR-dependent activation of αβ CD8^+^ T cells. The goal of this study was to characterize the ligandome displayed by HLA-E following infection with *Mycobacterium tuberculosis* (Mtb) using an in-depth mass spectrometry approach. Here we identified 28 Mtb ligands derived from 13 different source proteins, including the Esx family of proteins. When tested for activity with CD8^+^ T cells isolated from sixteen donors, nine of the ligands elicited an IFN-γ response from at least one donor, with fourteen of 16 donors responding to the Rv0634A_19-29_ peptide. Further evaluation of this immunodominant peptide response confirmed HLA-E restriction and the presence of Rv0634A_19-29_-reactive CD8^+^ T cells in the peripheral blood of human donors. The identification of an Mtb HLA-E ligand that is commonly recognized may provide a target for a non-traditional vaccine strategy.

## Introduction

Tuberculosis (TB), caused by *Mycobacterium tuberculosis* (Mtb), remains a leading cause of infectious mortality worldwide, accounting for 9.6 million new cases and 1.5 million deaths in 2014 (WHO, Global Tuberculosis Report 2015). Although the incidence rates worldwide are slowly declining, treatment of active cases alone is not likely to lead to the eradication of TB [1]. In contrast, vaccines that either prevent infection or prevent progression once infected can have a profound impact. The need for such a vaccine is further highlighted by the emergence of highly drug-resistant strains of Mtb. Development of an improved vaccine depends upon the identification of true correlates of protective immunity and an improved understanding of the mechanisms by which infection with Mtb is either prevented or contained. Despite the large numbers of those with TB worldwide, our immune system is actually remarkably successful in containing Mtb infections. Of those who are exposed to Mtb, approximately 50% go on to convert their TST, and of those who convert their TST, only 2–5% will develop active disease [2–4]. Consequently, we have focused on developing an improved understanding of the mechanisms by which the human immune system can recognize intracellular infection with Mtb.

While CD4^+^ T cells and proinflammatory cytokines such as IFN-γ and TNF-α are essential in the control of Mtb [5, 6], vaccination strategies targeting these responses have not necessarily proven to be protective. We and others have postulated that CD8^+^ T cells, through their direct recognition of the infected cell, could play a unique role in a protective immune response. Classically restricted CD8^+^ T cells are characterized by their activation via peptides presented in the context of the highly polymorphic HLA-Ia molecules. In contrast, non-classically restricted CD8^+^ T cells are characterized by their dependence on molecules that are not restricted to a specific donor. We and others have shown that non-classical CD8^+^ T cells restricted by HLA-E, MR1, and CD1 molecules can recognize antigens presented by Mtb [7–11]. These T cells can be found in high numbers in the blood and tissues, where they recognize intracellular infection with Mtb, including infected MHC class II negative cells, and have effector capacity associated with the control of Mtb (reviewed in [11]). Examples include MAIT cells, which recognize Vitamin B metabolites presented by MR1 molecules [7, 12], CD1a-c restricted cells, which recognize self and pathogen-derived lipids on CD1a-c molecules, and iNKT cells, which recognize lipid and glycolipid molecules presented by CD1d molecules [9]. Although CD8^+^ T cells can recognize Mtb-infected cells via HLA-E [8], little is known about the ligand(s) that are processed and presented for HLA-E in the context of intracellular infection. This molecule displays a very limited polymorphism across all populations [13] and is not down-regulated with HIV infection [14] and thus has the potential to be a broadly applicable vaccine target. In support of HLA-E as a promising vaccine target, the Picker group recently demonstrated that CD8^+^ T cells elicited by cytomegalovirus vector vaccination of rhesus macaques were restricted by HLA-E, which presented a diverse range of SIV peptides [15]. Additionally, HLA-E-restricted T cells are capable of both Th1- and Th2-like responses [16–18], further demonstrating their potential for broad functional utility.

As with other non-classical Class I molecules, HLA-E presents both self and pathogen-derived antigens to CD8^+^ T cells [8, 19–21]. The self-derived ligands recognized through the T-cell receptor (TCR) include peptides derived from the signal sequences of classical class I molecules [22]. Although HLA-E is known to present pathogen-derived antigens from bacterial pathogens including Mtb [8, 21], specific ligands generated during infection remain largely unidentified. The Ottenhoff group successfully used in silico predictions to predict HLA-E ligands from Mtb [16]. Here, we sought to directly identify HLA-E ligands processed and presented by Mtb-infected cells. HLA-E*01:03 was purified from cells infected with Mtb and the peptide ligands eluted from HLA-E were identified by two-dimensional LCMS. One of the epitopes, a peptide derived from the conserved hypothetical Mtb protein Rv0634A, was strongly and broadly recognized by CD8^+^ T cells from donors with Mtb infection, latent Mtb infection (LTBI), as well as healthy donors. The response was blocked with an anti-Class I antibody and could be elicited using antigen presenting cells that did not express matched HLA-A, B, or C alleles. Furthermore, CD8^+^ T cells expanded with the peptide could be stained with an HLA-E tetramer. As HLA-E is highly conserved and not down-regulated in HIV, these Mtb-derived HLA-E epitopes could be used in novel vaccination strategies targeting donor-unrestricted T cells.

## Materials and methods

### Human subjects and ethics statement

This study was conducted according to the principles expressed in the Declaration of Helsinki. Samples were collected under protocols approved by the Institutional Review Board (IRB) at Oregon Health Science University (OHSU, IRB0000186). All study participants were adults. Written informed consent was obtained for all participants and samples were assigned a random three-digit code by the study coordinator. Peripheral blood mononuclear cells (PBMC) were obtained by apheresis from healthy, LTBI, and Mtb-infected adult donors recruited from OHSU as previously described [23], or via IRB-approved advertisement at local TB clinics. Uninfected individuals were defined as healthy individuals with a negative tuberculin skin test and no known risk factors for infection with Mtb. Individuals with LTBI were defined as healthy individuals with a positive tuberculin skin test and no symptoms and signs of active TB. Individuals with active TB were diagnosed by the TB Controller for Multnomah or Washington Counties in Oregon, United States, and confirmed by positive sputum culture for Mtb.

### Bacteria and cells

The H37Rv strain of *M*. *tuberculosis* expressing GFP was a kind gift from David Sherman (Seattle Biomedical Research Institute, WA). Bacteria were grown in Middlebrook 7H9 broth supplemented with Middlebrook ADC (Fisher), 0.05% Tween-80, and 0.5% glycerol. Before infection, bacteria were passaged through a tuberculin syringe to obtain a single cell suspension. For infection of the bioreactor, 10 ml of a culture containing 5e9 bacteria was added to 1L of RPMI 1640 with 10% heat inactivated FBS and fed to the bioreactor over the course of 24 hours.

A549 cells were obtained from ATCC (CCL-185) and cultured in F12K with 10% heat inactivated FBS. U373 cells were a kind gift from the lab of Dr. Lewis Lanier (University of California, San Francisco) and were cultured in DMEM with 10% heat inactivated FBS. Monocyte-derived dendritic cells (DC) were prepared from PBMC as described [24]. Briefly, PBMC were resuspended in 2% heat-inactivated human serum (HuS) in RPMI and allowed to adhere to a T-75 (Costar) flask at 37C for 1 hr. After gentle rocking, non-adherent cells were removed and 10% heat-inactivated HuS in RPMI containing 10 ng/ml IL-4 (Immunex) and 30 ng/ml GM-CSF (Immunex) was added to the adherent cells. After 5 days, cells were harvested with cell-dissociation medium (Sigma-Aldrich) and used as indicated in assays. CD8^+^ T cells were isolated from PBMC by positive selection using CD8 antibody-coated magnetic beads per the manufacturer’s instructions (StemCell Technologies). The D160 1–23 HLA-E restricted CD8^+^ T cell clone was expanded and maintained as previously described [8].

### Transfection and purification of the sHLA-E ligand pool

**Fig 1:**
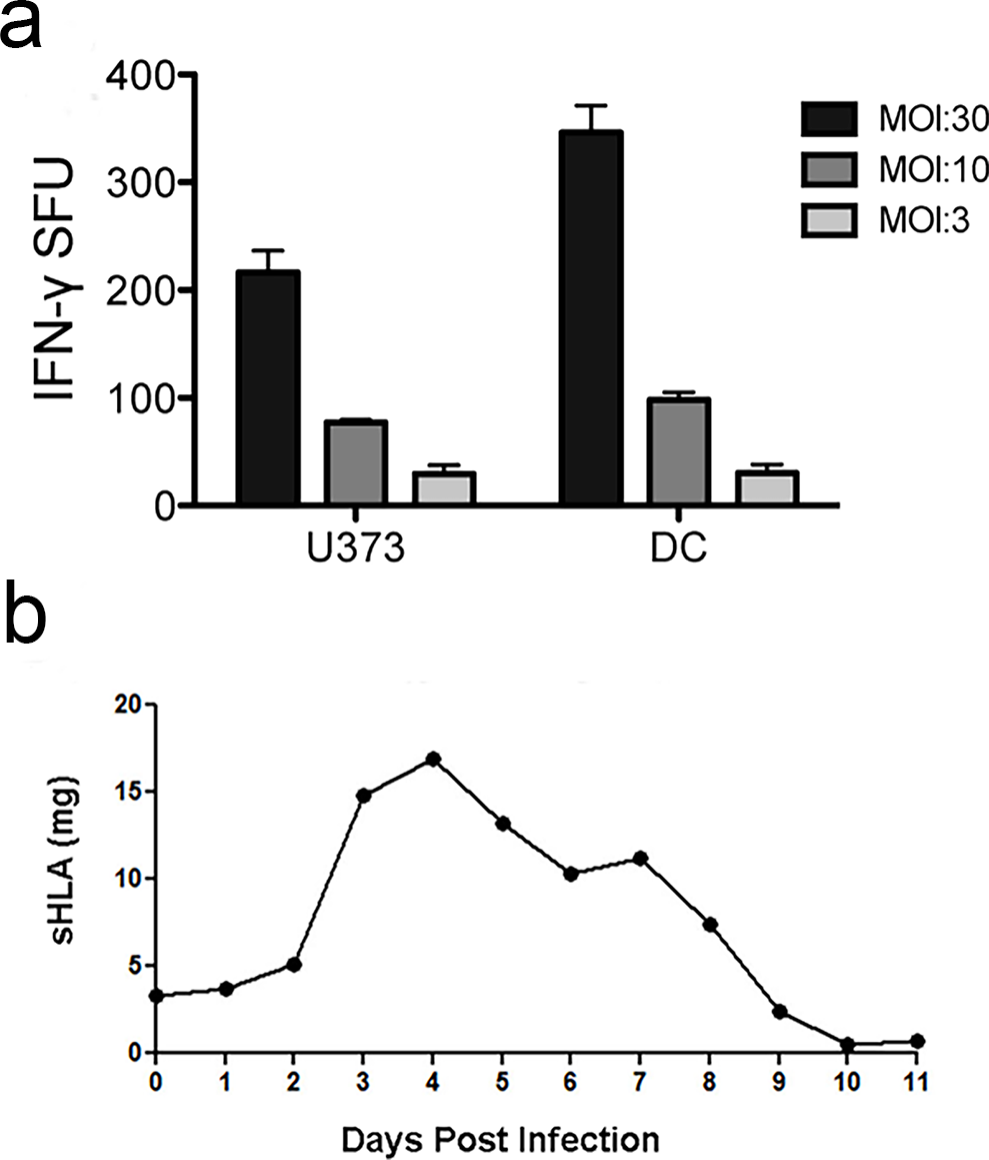
Production of soluble HLA-E from Mtb-infected cells. A) U373 or DC were infected with the H37Rv strain of Mtb (multiplicity of infection (MOI):30, 10, or 3) for 18 hours, then used as antigen presenting cells in an ELISPOT assay with IFN-γ production by D160 1–23, an HLA-E restricted CD8^+^ T cell clone, as the readout. B) U373 cells expressing sHLA-E, and grown to high density in a hollow-fiber bioreactor, were infected with Mtb. sHLA-E was measured in the cell supernatant using ELISA.

To select a cell line for soluble HLA-E production, we tested fifteen human cell lines to determine how well each cell type was able to present Mtb-derived antigens to a human HLA-E restricted T cell clone compared to primary human dendritic cells (DC), as well as how efficiently each cell type produced recombinant HLA-E molecules. The HLA-E restricted T cell clone D160 1–23 was able to make IFN-γ in response to Mtb-infected U373 cells at levels similar to DC (Fig 1A), and U373 cells produced sufficient quantities of soluble HLA-E molecules for downstream mass spectrometry analyses. It was previously demonstrated that there is little difference between the peptide repertoire loaded on soluble MHC Class I versus full length molecules [25]. However, we also verified that the Mtb ligands generated in the context of soluble MHC Class I produced by infected cells would be reflective of Mtb antigens presented in a physiologically relevant context. To do this, we analyzed ligands from Mtb-infected cells expressing soluble HLA-B44 and found several of the known immunodominant ligands derived from the Mtb CFP10 protein (IEDB reference #1028103) [26].

After validating our model system, U373 cells were transfected with a previously described soluble form of HLA-E*01:03 [27]. Cells were electroporated with a construct where the cytosolic and transmembrane domains of HLA-E*01:03 were replaced with a purification tag from the VLDL receptor in the mammalian expression vector pcDNA3.1(-). Stable transfectants were obtained after drug selection and subcloned by limiting dilution. sHLA-E was measured in the cell supernatant with W6/32 (anti-pan HLA class I) and 3D12 (anti-HLA-E) capture ELISA using an anti-β2 microglobulin antibody as a detector. The best producing clone was selected and grown to high-density in a hollow fiber bioreactor. 46.6 mg of sHLA-E from uninfected cells was collected before infecting the cells with 5e9 CFU of Mtb strain H37Rv GFP. Every day after infection, a cell sample was taken and cells infected with GFP bacteria were enumerated using florescence microscopy. At least 50% of viable cells in each cell sample were infected with Mtb. Mtb infected and uninfected sHLA-E was purified using affinity chromatography with an anti-VLDLr antibody. sHLA was eluted from the affinity column with 0.2M acetic acid and further acidified to 10% acetic acid. Ligands were eluted from the HLA complex with a boil at 75° C for 15 minutes. The ligand pool was isolated from the alpha chain and β-2m with 3kDa cut-off ultrafiltration. The isolated ligand pool was lyophilized and resuspended in 10% acetic acid for HPLC.

### HPLC and LCMS analysis of ligand pool

Class I HLA ligands are complex and have similar lengths and biochemistry, so a two-dimensional HPLC separation was utilized for the best sensitivity, as recently described [28]. Briefly, the ligand pool was loaded onto a reverse phase 2mm by 150mm Jupiter 4 μm Proteo (Phenomenex) HPLC column with a Michome Paradigm MG4 HPLC (Michome). After a desalt wash, the peptides were eluted from the column at 160μl/min with a gradient of acetonitrile and water at pH 10 (2–10% for 2 min, 10–60% for 60 min). Eluted peptides were collected into 1 minute fractions along the gradient. Next, fractions from 22–60 minutes were analyzed individually using nano-LCMS described in detail previously [28]. Eluted peptides were ionized using a Nanospray III ion source (AB Sciex) and analyzed on an AB Sciex 5600 Triple TOF in DDA acquisition mode. For the MS quantitation experiments, twenty percent of each fraction was analyzed the same way except the data was collected in SWATH mode using a mass range of 300–1150 m/z and a SWATH window of 25 m/z for a total cycle time of 3.36 seconds.

### MS data analysis

For the *de novo* identification of HLA-E ligands, data-dependent acquisition (DDA) spectra from both infected and uninfected cells were analyzed using PEAKS 7.0 and Mascot. For both algorithms, three databases were used: the *M*. *tuberculosis* NCBInr for Mtb ligands, *H*. *sapiens* NCBInr for non-HLA human ligands, and a custom HLA sequence database for leader peptide sequences. For PEAKS and Mascot, n-terminal acetylation, deamidation of N and Q, oxidation of M,W,H, pyro-glu from Q, and sodium adducts of D,E and c-terminus were used as variable modifications. All positive identifications were made using a 1% FDR at a mass error tolerance of 50 ppm with most positive identifications within 25 ppm of theoretical mass. For all Mtb sequences, a synthetic peptide (Genemed Synthesis) with the corresponding sequence was analyzed under the same LCMS conditions. Only peptides that matched the fragment pattern, were within 50 ppm, and eluted within 5 min of the synthetic were further characterized.

For the MS quantitation experiment, a spectral library was generated using the ligands from both infected and uninfected cells. The spectral library consisted of only ligands identified at a 1%FDR by PEAKS and Mascot. Fragment ion information for each peptide was extracted from a *H*. *sapiens* Protein Pilot 4.5 search of the DDA data with no score threshold set. SWATH data for all first-dimension fractions from both infected and uninfected cells was searched using the spectral library described above with Peakview 2.0 with the SWATH 2.0 microapp. Intensity values were generated using the sum of the top 5 fragment ions within 50 ppm and a RT window of 5 minutes. Intensities from modifications and different charge states were summed for each distinct peptide. To eliminate false-positives the intensity data for every peptide in every first dimension fraction was only analyzed further if the intensity value was above 10000 counts. For the infected and uninfected samples, the thresholded intensity values for each first-dimension fraction were summed. Next, to account for total peptide quantity, the intensity data was normalized to the TIC. The log_10_ fold increase or decrease was then calculated for each peptide.

### ELISPOT assay

The IFN-γ ELISPOT assay was performed as described previously [8]. CD8^+^ T cells isolated as described above were used a source of responder T cells and tested in duplicate at 250,000 cells/well. In some cases, autologous DCs (20,000 cells/well) were used as antigen presenting cells (APC), In other assays, U373 cells or HLA-mismatched A549 cells were used. Peptides were synthesized by Genemed Synthesis and resuspended in DMSO at a concentration of 20mg/ml. DC or A549s were pulsed with peptide (5ug/ml final concentration) and added to the assay. U373 cells were infected with H37Rv-GFP at MOI:3,10, or 30. T cells (250K/well) were incubated with DC or A549 cells, or for U373 cells, the HLA-E restricted human T cell clone, D160 1–23, in the presence peptide or infection with Mtb. Negative and positive controls were included in each assay and consisted of wells containing T cells and A549/DCs without antigen, or with inclusion of phytohemagglutanin (PHA, 10ug/ml; EMD Biosciences). For all assays, responding T cells were incubated with APC overnight. A positive ELISPOT response was defined as one in which the peptide specific response was at least two standard deviations above the background control. The peptide specific T cell response was then determined by subtracting the mean of the background from the positive response.

### Generation of T cell line and tetramer staining

Dendritic cells (DC) were prepared from D481 PBMC as described above and plated in ultra-low adherence plates at 1e5/ml. DC were incubated with Rv0634a_19-29_ peptide at 2ug/ml final concentration for 24 hours at 37 degrees C. Autologous CD8^+^ T cells were isolated from D481 PBMC as described above and added to the peptide pulsed DC at 1e6/ml with 5ng/ml IL-2. After 6 days, CD8^+^ T cells were harvested from wells containing peptide pulsed DC and labeled with CFSE. 1e6 CFSE-labeled CD8^+^ T cells were then transferred to an ultra-low adherence plate containing 1e5 Rv0634a_19-29_ peptide-pulsed autologous macrophages and incubated with 5ng/ml IL-2 for 6 days. After 6 days, the CD8^+^ T cells were sorted on CFSE staining. CFSE-dim cells were collected and tested for response to the Rv0634a_19-29_ peptide by IFN-γ ELISPOT assay. The CD8^+^ T cell line expanded from D481 as described above was also incubated with either a control HLA-B15 tetramer (recognizing a peptide from the Mtb protein CFP10_75-83_) or the Rv0634a_19-29_-HLA-E tetramer (HLA-E*1:03 coupled to PE, NIH Tetramer Core) at a 1:50 dilution for 1 hour at 37 degrees C. The cells were then stained with LIVE/DEAD Fixable Dead Cell Stain Kit (Invitrogen) and subsequently stained with antibodies to CD8 (APC Cy7), CD3 (BV650), and CD4 (BV421) for 30 min at 4 degrees C.

## Results

### Production of sHLA-E from Mtb infected cells

At present, HLA-E ligands presented by the Mtb-infected cell remain largely unknown. Because of the size and complexity of the Mtb genome, bioinformatic identification of HLA class I (HLA I) epitopes is challenging and often does not cover the entire genome. We have previously pioneered the use of stably transfected cells expressing soluble HLA molecules to define both exogenous and endogenous ligands from virally infected cells [27–29]. This strategy has not been used for an intracellular bacterial pathogen such as Mtb. To identify Mtb specific HLA-E ligands we utilized a cell infection model where soluble HLA-E*01:03 was isolated from cells infected with Mtb. The HLA-E*01:03 allele is present in approximately half of the population, and has a single amino acid difference from the HLA-E*01:01 allele. U373 cells, which are infected with Mtb and present Mtb antigen to HLA-E restricted T cells (Fig 1A), and produce sufficient amounts of soluble HLA Class I molecules, were stably transfected with soluble HLA-E and grown to high-density in a hollow-fiber bioreactor. The cells reached peak sHLA-E production 48 days after seeding, resulting in the production of 46.6mg of sHLA-E from uninfected U373 cells. On day 48, the cells were infected with Mtb-H37Rv expressing GFP. Strikingly, 3 days after infection there was a large spike in sHLA-E production that was followed by a steady decrease over time corresponding to the expected reduction in cell viability (Fig 1B). The 11 day production run resulted in a total of 89.6 mg of sHLA-E from Mtb-infected cells.

### Identification of class I HLA leader peptides eluted from sHLA-E molecules

**Fig 2:**
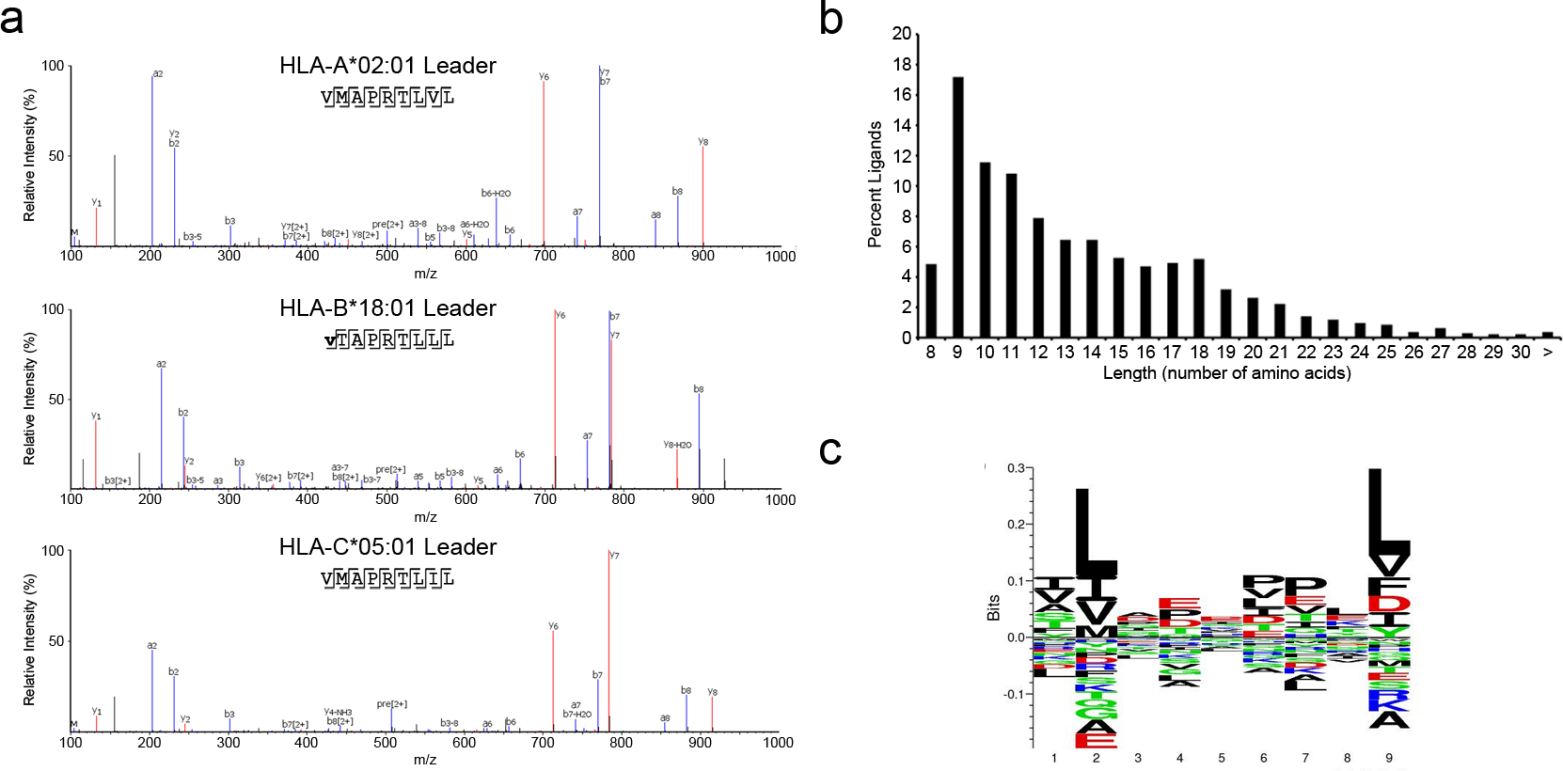
Class I leader peptides eluted from sHLA-E molecules. A) Representative annotated MS2 fragment ion spectra for the identified leader peptides from HLA-A*02:01, HLA-B*18:01, and HLA-C*05:01 that were eluted from purified sHLA-E. B) Length distribution of all non-redundant HLA-E ligands identified combined from Mtb infected and uninfected cells. C) Peptide binding motif of nonamer peptide sequences eluted from HLA-E. Motif was generated using Seq2Logo.

Like other HLA molecules, cell surface expression of HLA-E depends on the presence of a suitable peptide [22, 30]. HLA-E is known to present leader peptides derived from HLA I molecules such as HLA-A, B, and C as well as HLA-G [22, 30]. We predicted the sHLA-E would likewise bind HLA I leader peptides from HLA-A*02:01, HLA-B*18:01, and HLA-C*05:01, the HLA I haplotype of the U373 cells. Based on this haplotype, we expect there to be two high affinity leader peptides; VMAPRTLVL (HLA-A2), VMAPRTLIL (HLA-C5) and one low affinity peptide VTAPRTLLL (HLA-B18) generated from these HLA alleles [31]. Analysis of the ligand pool from both Mtb infected and uninfected cells revealed the presence of both high affinity leader peptides. The low affinity HLA-B18 leader peptide could be found, but it was both low abundance and n-terminally acetylated. The annotated fragment spectra for all leader peptides are shown in Fig 2A. In addition to the expected leader peptides, we observed multiple variants of these leader peptides (Table 1). In both HLA-A2 and HLA-C5, there were unmodified and modified length variants. For the HLA-A2, -B18, and -C5 peptides we found a total of 10, 2, and 9 variants respectively. For the HLA-A2, and -C5 peptides the P2 methionine was oxidized, while leader peptides from all allomorphs had at least one n-terminal acetylation. The leader peptides also varied in length from 7 to 11 amino acids. Together these results demonstrate that we are able to detect the expected HLA I leader peptides as well as multiple length and modification variants among the ligands associated with HLA-E expressed in U373 cells.

**Table 1:** Leader sequences associated with soluble HLA-E.

Allomorph	Modified Sequence	Stripped Sequence	m/z	RT	Length	Intensity (Mtb)	Intensity (UI)
A0201	VM(+15.99)APRTLVL	VMAPRTLVL	508.3017	29.14	9	4.25E+08	5.00E+08
	VMAPRTLVL	VMAPRTLVL	500.3066	32.16	9	8.62E+07	2.92E+08
	VM(+15.99)APRTLV	VMAPRTLV	451.7606	26.28	8	1.80E+05	4.42E+06
	V(+42.01)MAPRTLV	VMAPRTLV	464.7668	40.52	8	B.T.	4.40E+05
	MAPRTLVL	MAPRTLVL	450.7628	32.62	8	1.01E+05	2.46E+05
	M(+42.01)AVMAPRTLVL	MAVMAPRTLVL	622.3673	42.84	11	8.60E+04	1.61E+05
	M(+15.99)APRTLVL	MAPRTLVL	458.7669	30.54	8	2.16E+05	1.39E+05
	VMAPRTLV	VMAPRTLV	443.7597	27.82	8	4.09E+04	9.57E+04
	APRTLVL	APRTLVL	385.2513	29.42	7	1.28E+05	8.38E+04
	VM(+15.99)APRTL	VMAPRTL	402.2261	24.21	7	1.29E+04	1.37E+04
	VMAPRTLVLLL	VMAPRTLVLLL	613.3701	66.54	11	B.T.	B.T.
B1801	V(+42.01)TAPRTLLL	VTAPRTLLL	513.3088	54.69	9	B.T.	B.T.
	R(+42.01)VTAPRTLLL	RVTAPRTLLL	591.3685	63.19	10	4.17E+04	7.64E+04
C0501	VMAPRTLIL	VMAPRTLIL	515.3138	35.93	9	1.08E+08	6.09E+08
	VM(+15.99)APRTLIL	VMAPRTLIL	507.3148	38.85	9	3.03E+08	4.01E+08
	V(+42.01)MAPRTLIL	VMAPRTLIL	515.3138	35.93	9	1.54E+06	5.27E+06
	VMAPRTLI	VMAPRTLI	528.3139	48.92	9	8.20E+04	3.97E+06
	VM(+15.99)APRTLI	VMAPRTLI	450.7709	31.46	8	7.92E+04	2.87E+06
	RVMAPRTLIL	RVMAPRTLIL	458.7679	28.88	8	1.33E+06	2.42E+06
	RVM(+15.99)APRTLIL	RVMAPRTLIL	585.3442	65.46	10	1.94E+06	1.70E+06
	R(+42.01)VMAPRTLIL	RVMAPRTLIL	593.3468	53.1	10	1.40E+05	1.17E+05
	MAPRTLIL	MAPRTLIL	606.3652	59.41	10	5.28E+04	9.99E+04
	V(+42.01)MAPRTLI	VMAPRTLI	457.777	34.48	8	B.T.	3.50E+04

(+15.99) indicates oxidized methionine

(+42.01) indicates acetylated N-terminus

B.T. indicates below threshold

UI indicates uninfected cells

In addition to the leader peptides we observed a combined total of 1263 host cell-derived non-leader ligands from 324 source proteins from both infected and uninfected cells. Ligands ranged from 8 to 33 amino acids in length with nonamers being the most common length (Fig 2B). Among the nonamers, we observed an HLA-A2-like motif (Fig 2C). Thus, consistent with previous reports [15, 32], there are a large number of host-derived HLA-E ligands that are not HLA I leader peptides, and have a similar peptide binding motif to HLA-A*02:01.

### Mtb infection alters the HLA-E host ligand repertoire and results in a minor Mtb-specific ligand pool

**Fig 3:**
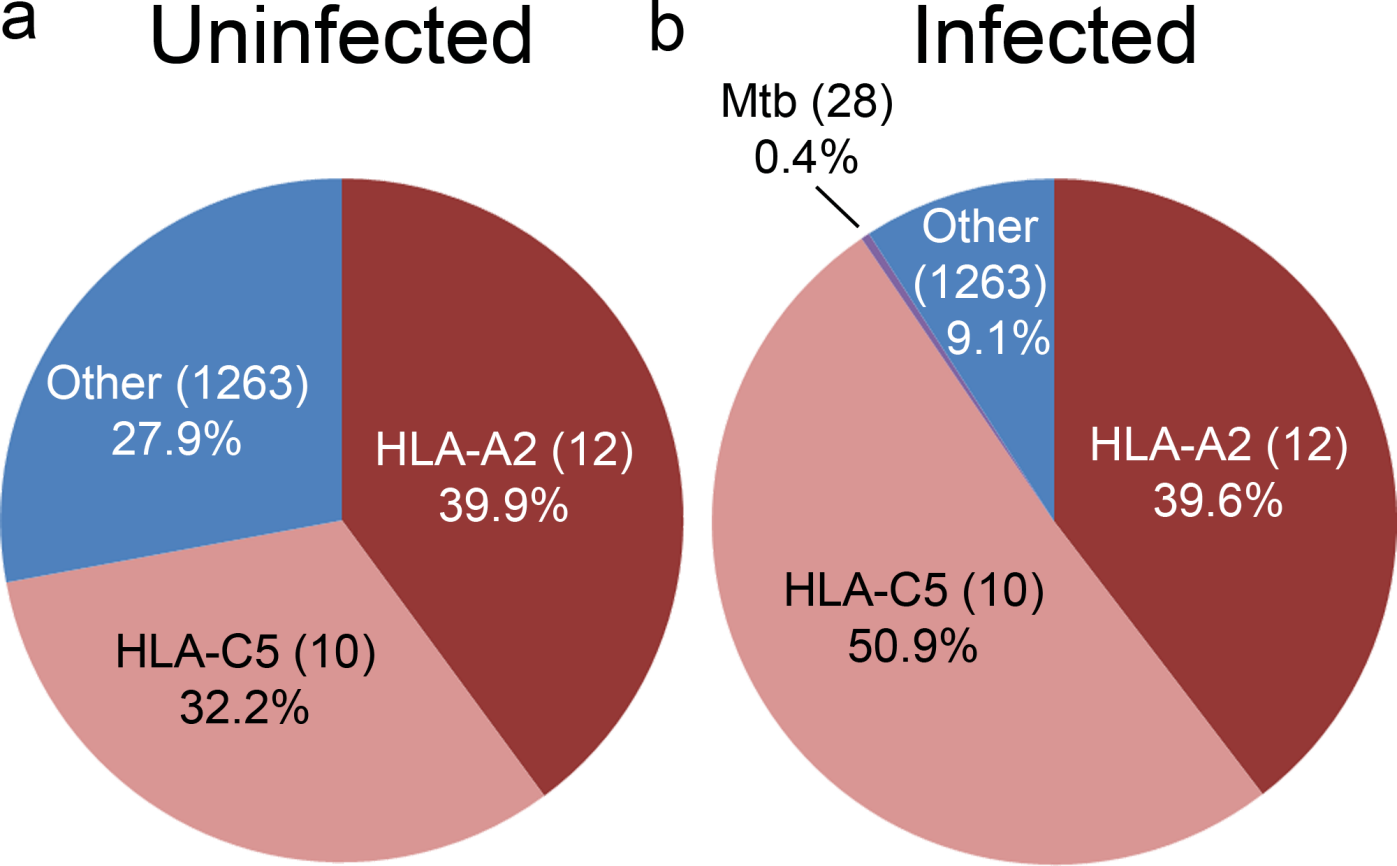
Mtb infection alters the HLA-E host ligand repertoire and results in a minor Mtb-specific ligand pool. Percentage of total ion intensity as determined in the materials and methods section of indicated HLA-E ligand groups from uninfected (A) or Mtb infected cells (B).

In prior work, we found that viral infection can alter the repertoire of host-derived HLA-I ligands [33]. To assess the impact of Mtb infection on host-derived HLA-E ligands, we compared the HLA-E host ligand repertoire from uninfected and Mtb infected cells. Using data independent acquisition (SWATH), we obtained intensity information for all of the identified host and Mtb ligands in both infected and uninfected cells. As expected, the most predominant ligands in both infected and uninfected cells were the previously described high-affinity HLA-A2 and HLA-C5 leader peptides (Fig 3A). While the HLA-B18 leader peptide was detectable in both infected and uninfected cells it accounted for 0.004% and 0.003% of the total ligand intensity respectively. In the uninfected cells, the HLA-A2 and HLA-C5 leader peptides and variants accounted for 39.9% and 32.2% of the ligand intensity respectively, with the remaining 27.9% of the intensity coming from the 1263 minor ligands. After infection, the HLA-C5 leader peptide variants became more predominant, accounting for 50.9% of the ligand intensity. Surprisingly, the HLA-A2 was detected at the same levels as the uninfected cells (39.6%) and the overall intensity of the minor ligands was decreased (9.1%) (Fig 3B**)**. The Mtb-derived ligands, discussed below, were also a minor ligand category, accounting for 0.4% of the total ligand intensity. Thus, after Mtb infection the HLA-C5 leader peptide displaces the host-cell minor ligand pool, with Mtb-derived ligands accounting for a small fraction of the total ligand pool.

### Characterization of Mtb-derived ligands from sHLA-E molecules

**Fig 4:**
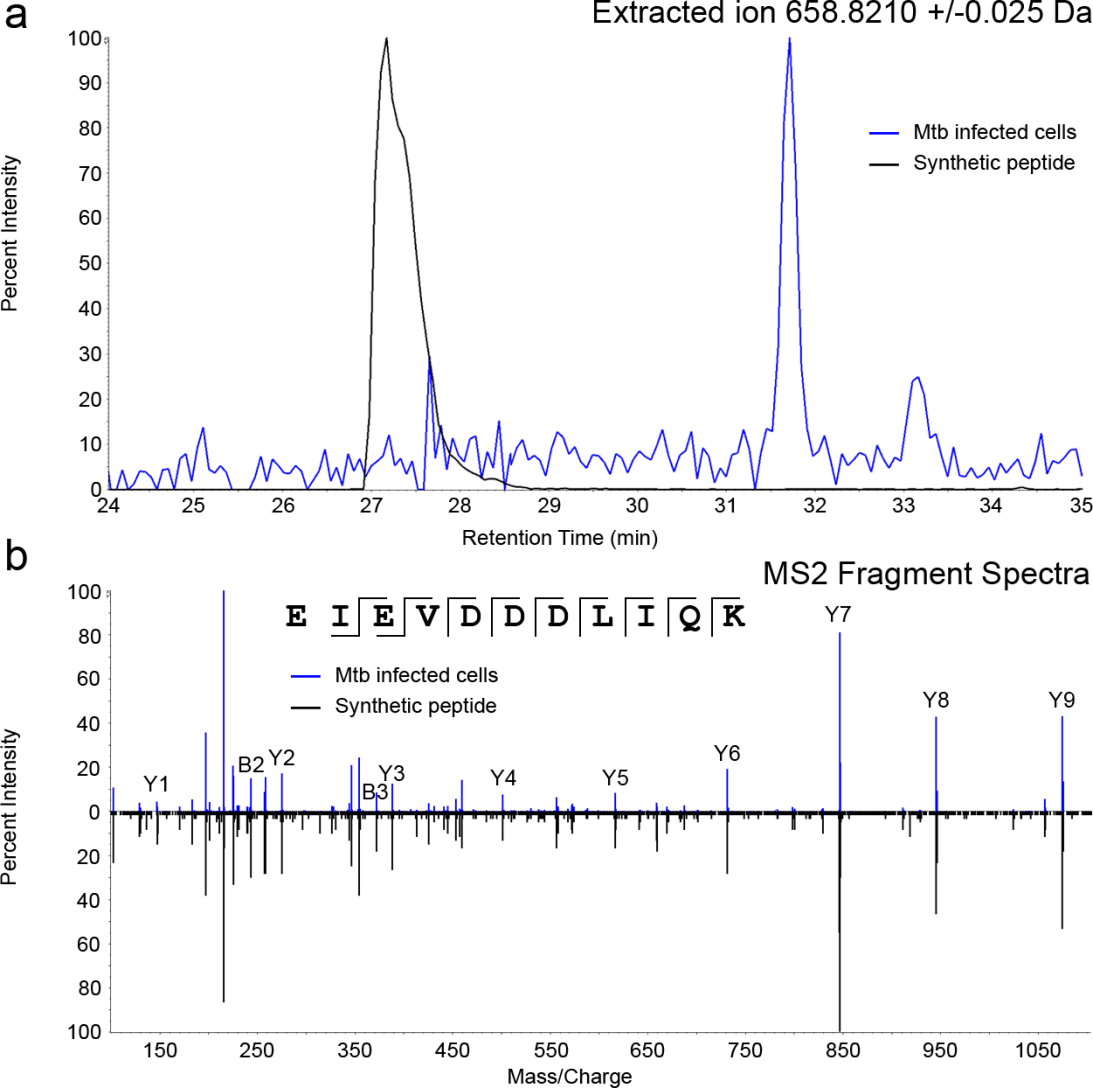
An Mtb ligand derived from Rv0634A is eluted from sHLA-E molecules. A) Extracted ion chromatogram of ion 658.8210 (EIEVDDDLIQK) in Mtb infected ligand pool (blue trace) and synthetic peptide (black trace). B) Annotated MS2 fragment ion spectra for the identified Mtb peptide EIEVDDDLIQK in Mtb infected ligand pool (blue trace) and synthetic peptide (black trace).

We identified 28 Mtb-derived sequences originating from 13 proteins (Table 2) in the HLA-E ligand pool from Mtb infected cells. To confirm the sequence assignment, all 28 peptides were synthesized and subjected to the same LCMS conditions as the eluted ligands. As a representative example, extracted ion chromatogram and the annotated fragment spectra from the Mtb-derived ligand EIEVDDDLIQK from the Rv0634A (Rv0634A_19-29_) source protein, with the corresponding synthetic peptide are shown in Fig 4 demonstrating that the sequence was correctly identified. In this manner, we confirmed that all 28 sequences were correctly assigned. Mtb ligands ranged in length from 8 to 20 amino acids in length, consistent with what we observed in the host ligand repertoire. Most of the ligands were unmodified, however two ligands contained an oxidized methionine and one ligand contained an N-terminal acetylation (Table 2). Half of the eluted ligands (14/28) originated from known antigenic ESX operon proteins including EspA, ESAT-6, EsxG, EsxH and PE5. EsxG was the most abundantly sampled source protein with 6 ligands. Additionally, we identified two previously reported HLA-A2 restricted ligands from ESAT-6 (SLLDEGKQSL) [34] and EsxG (LLDAHIPQL) [26]. Other Mtb source proteins included ftsH, iniB, lpqI, mpa, rplA, Rv0435c, Rv0634A, and Rv3479. Thus, after Mtb infection, HLA-E binds and presents Mtb-derived peptides originating from both Esx and non-Esx operon proteins.

**Table 2:** Mtb peptides associated with sHLA-E.

Sequence	Gene	Source Protein	Locus Tag	Length	Start AA	End AA	Source Protein Length	1D Fraction	2D RT (min)	Obs. m/z	Obs. z	Obs. Mass	Δ ppm	PEAKS Score	Mascot Score	SWATH Intensity
ATNRPDILD	ftsH	Membrane-bound protease FtsH	Rv3610c	9	307	315	760	24	21.6	507.7673	2	1013.5140	6	41.4	48.3	1.51E+05
TNRPDILD	ftsH	Membrane-bound protease FtsH	Rv3610c	8	308	315	760	23	24.2	472.2327	2	942.4770	-28	35.3	39.6	B.T.
ATNRPDLID	Rv0435c	Putative conserved ATPase	Rv0435c	9	607	615	728	24	21.6	507.7673	2	1013.5140	6	41.4	48.3	1.51E+05
TNRPDLID	Rv0435c	Putative conserved ATPase	Rv0435c	8	608	615	728	23	24.2	472.2327	2	942.4770	-28	35.3	39.6	B.T.
AERAPVEADAGGGQKVLVRN	espA	ESX-1 secretion-associated protein A, EspA	Rv3616c	20	371	391	392	32	27.3	559.5590	4	2234.2129	-3	84.4	108.3	B.T.
STEGNVTGMFA	esxA	6 kDa early secretory antigenic target EsxA (ESAT-6)	Rv3875	11	85	95	95	30	36.4	557.2463	2	1112.4807	-2	48.2	64.0	3.64E+05
STEGNVTGmFA	esxA	6 kDa early secretory antigenic target EsxA (ESAT-6)	Rv3875	11	85	95	95	26	27.0	565.2434	2	1128.4758	-3	44.8	-	8.72E+04
SLLDEGKQSL	esxA	6 kDa early secretory antigenic target EsxA (ESAT-6)	Rv3875	10	27	36	95	27	31.8	545.2885	2	1088.5713	-8	54.9	76.2	2.07E+05
LLDEGKQSL	esxA	6 kDa early secretory antigenic target EsxA (ESAT-6)	Rv3875	9	28	36	95	28	25.2	501.7712	2	1001.5394	-11	47.1	89.8	1.51E+05
TLLDVAQANLGEAAGTYV	esxG	ESAT-6 like protein EsxG	Rv0287	18	67	84	97	36	51.8	602.6515	3	1804.9207	7	70.7	134.5	3.04E+05
NLGEAAGTYV	esxG	ESAT-6 like protein EsxG	Rv0287	10	75	84	97	27	31.9	497.7424	2	993.4767	-7	46.5	61.3	2.01E+04
SLLDAHIPQL	esxG	ESAT-6 like protein EsxG	Rv0287	10	2	11	97	36	43.7	553.8143	2	1105.6130	1	57.1	80.4	1.22E+05
LLDAHIPQL	esxG	ESAT-6 like protein EsxG	Rv0287	9	3	11	97	37	39.5	510.2991	2	1018.5811	3	53.9	64.4	B.T.
SLLDAHIPQ	esxG	ESAT-6 like protein EsxG	Rv0287	9	2	10	97	32	33.0	497.2689	2	992.5291	-6	48.6	81.5	3.39E+06
LLDAHIPQ	esxG	ESAT-6 like protein EsxG	Rv0287	8	3	10	97	33	28.4	453.7538	2	905.4970	-4	39.8	66.2	3.32E+04
IMYNYPAML	esxH	Low molecular weight protein antigen 7 EsxH	Rv0288	9	4	12	96	42	45.6	558.2668	2	1114.5190		35.6	32.1	3.73E+05
ImYNYPAmL	esxH	Low molecular weight protein antigen 7 EsxH	Rv0288	9	4	12	96	34	36.7	574.2602	2	1146.5090	-3	36.2	-	4.15E+04
GLIDIAPHQISSVAA	iniB	Isoniazid inductible gene protein IniB	Rv0341	15	33	47	479	37	38.6	746.4158	2	1490.8091	5	58.7	72.9	4.08E+05
HVGGILIGSDTDLT	lpqI	Probable conserved lipoprotein LpqI	Rv0237	14	87	100	388	31	37.4	699.3642	2	1396.7200	-4	65.7	127.2	2.01E+05
VGGILIGSDTDLT	lpqI	Probable conserved lipoprotein LpqI	Rv0237	13	88	100	388	31	42.4	630.8350	2	1259.6609	-4	56.7	69.1	2.04E+05
GGILIGSDTDLT	lpqI	Probable conserved lipoprotein LpqI	Rv0237	12	89	100	388	28	38.7	581.2955	2	1160.5925	-14	54.0	68.7	7.31E+05
AEIVAGGGPLPL	lpqI	Probable conserved lipoprotein LpqI	Rv0237	12	107	118	388	32	42.4	547.3130	2	1092.6179	-6	63.3	88.7	3.44E+05
PAILRPGRLD	mpa	Mycobacterial proteasome ATPase Mpa	Rv2115c	10	423	432	609	42	25.0	369.8908	3	1106.6560	-5	53.8	57.3	B.T.
RVVPEGLAAA	PE5	PE family protein PE5	Rv0285	10	4	13	102	31	28.7	491.7827	2	981.5607	-10	15.8	80.7	B.T.
AGADVVGSDDLIE	rplA	50S ribosomal protein L1 RplA	Rv0641	13	90	102	235	23	35.0	630.7905	2	1259.5881	-17	41.7	73.5	9.50E+04
ADVVGSDDLIE	rplA	50S ribosomal protein L1 RplA	Rv0641	11	92	102	235	23	34.2	566.7641	2	1131.5295	-14	41.9	65.2	6.76E+04
EIEVDDDLIQK	Rv0634A	hypothetical protein	Rv0634A	11	19	29	83	23	31.7	658.8214	2	1315.6506	-17	40.2	66.7	5.89E+05
**s**TIIAGALLLVL	Rv3479	Possible transmembrane protein	Rv3479	12	900	911	1020	50	64.8	613.3760	2	1224.7693	-26	35.2	-	B.T.

“m” indicates oxidize methionine

“s” indicates N-terminal acetylation

### Identified Mtb ligands are recognized by human Mtb-reactive CD8^+^ T cells

**Fig 5:**
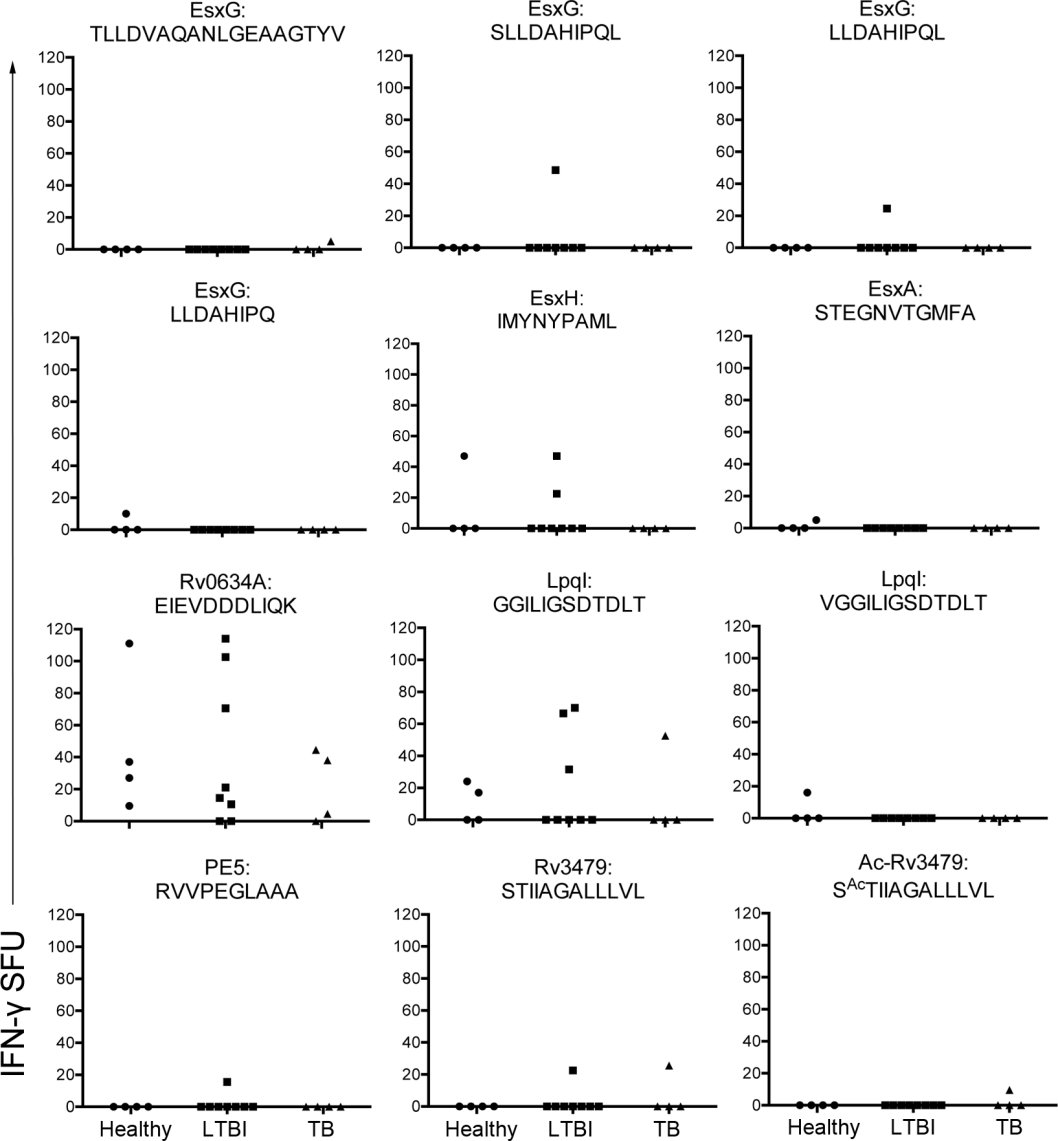
Mtb ligands are recognized by human Mtb-reactive CD8^+^ T cells. CD8^+^ T cells from four healthy, eight LTBI, and four Mtb-infected donors were incubated in an IFN-γ ELISPOT plate with autologous DC pulsed with each of the 28 validated Mtb peptides. Shown are the IFN-γ spot forming units for the 12 peptides for which at least one donor responded.

To determine whether or not Mtb-derived HLA-E ligands were immunogenic in the setting of infection with Mtb, each peptide was tested for its ability to elicit a response by human CD8^+^ T cells. CD8^+^ T cells isolated from PBMC from Mtb-infected (n = 4), LTBI (n = 8), or healthy (n = 4) donors were tested *ex vivo* with peptide-pulsed autologous DC used as APC for their ability to produce IFN-γ. Of the 28 peptides tested, 12 were recognized by at least one of the 16 donors tested (Fig 5**)**, and 14 of the 16 donors screened responded to at least one of the peptides (Table 3). The responses ranged from 5–114 IFN-γ ELISPOTs per 2.5e5 CD8^+^ T cells. CD8^+^ T cells from healthy donors responded to 6/28 peptides, with an average of 30.2(+/-30.6) IFN-γ spot forming units (SFU) per 2.5e5 CD8^+^ T cells; CD8^+^ T cells from LTBI donors responded to 7/28 peptides with an average of 42.6(+/-32.1) IFN-γ /SFU per 2.5e5 CD8^+^ T cells; and CD8^+^ T cells from Mtb-infected donors responded to 5/28 peptides with an average of 25.7(+/-20.8) IFN-γ /SFU per 2.5e5 CD8^+^ T cells.

**Table 3:** Responses from 250,000 CD8^+^ T cells *ex vivo* to peptide-pulsed autologous DC.

Peptide ID	Peptide Source Protein	Peptide Sequence	Responding Donors	IFNγ-SFU
**5**	EsxG_67-84_	TLLDVAQANLGEAAGTYV	D481 (TB)	5.5
**9**	LpqI_88-100_	VGGILIGSDTDLT	D519 (H)	16
**10**	EsxG_2-11_	SLLDAHIPQL	D454^a^ (LTBI)	48.5
**12**	EsxG_3-11_	LLDAHIPQL	D454^a^ (LTBI)	24.5
**15**	EsxA_85-95_	STEGNVTGMFA	D519 (H)	13
**16**	LpqI_89-100_	GGILIGSDTDLT	D698 (TB)	52.5
			D454^a^ (LTBI)	31.5
			D584 (LTBI)	66.5
			D603^a^ (LTBI)	70
**23**	Rv0634A_19-29_	EIEVDDDLIQK	D480 (TB)	14.5
			D481 (TB)	38
			D698 (TB)	44.5
			D454^a^ (LTBI)	102.5
			D557 (LTBI)	14.5
			D584 (LTBI)	70.5
			D603^a^ (LTBI)	114
			D673 (LTBI)	21
			D732 (LTBI)	10.5
			D444 (H)	27
			D497 (H)	37
			D519 (H)	111
			D520 (H)	9.5
**26**	Rv3479_900-911_	S(+42.01)TIIAGALLLVL^b^	D480 (TB)	9.5
**27**	Rv3479_900-911_	STIIAGALLLVL	D480 (TB)	25.5
			D732 (LTBI)	22.5
**28**	EsxG_3-10_	LLDAHIPQ	D519 (H)	10
**32**	EsxH_4-12_	IMYNYPAML	D584 (LTBI)	22.5
			D603^a^ (LTBI)	47
			D519 (H)	47
**34**	PE5_4-13_	RVVPEGLAAA	D564 (LTBI)	15.5

^a^Indicates donor is HLA-A2

^b^Peptide is acetylated at amino acid 900

TB: Active TB donor

LTBI: Latent TB donor

H: Healthy donor

### The CD8^+^ T cell response to Rv0634A_19-29_ is restricted by HLA-E

**Fig 6:**
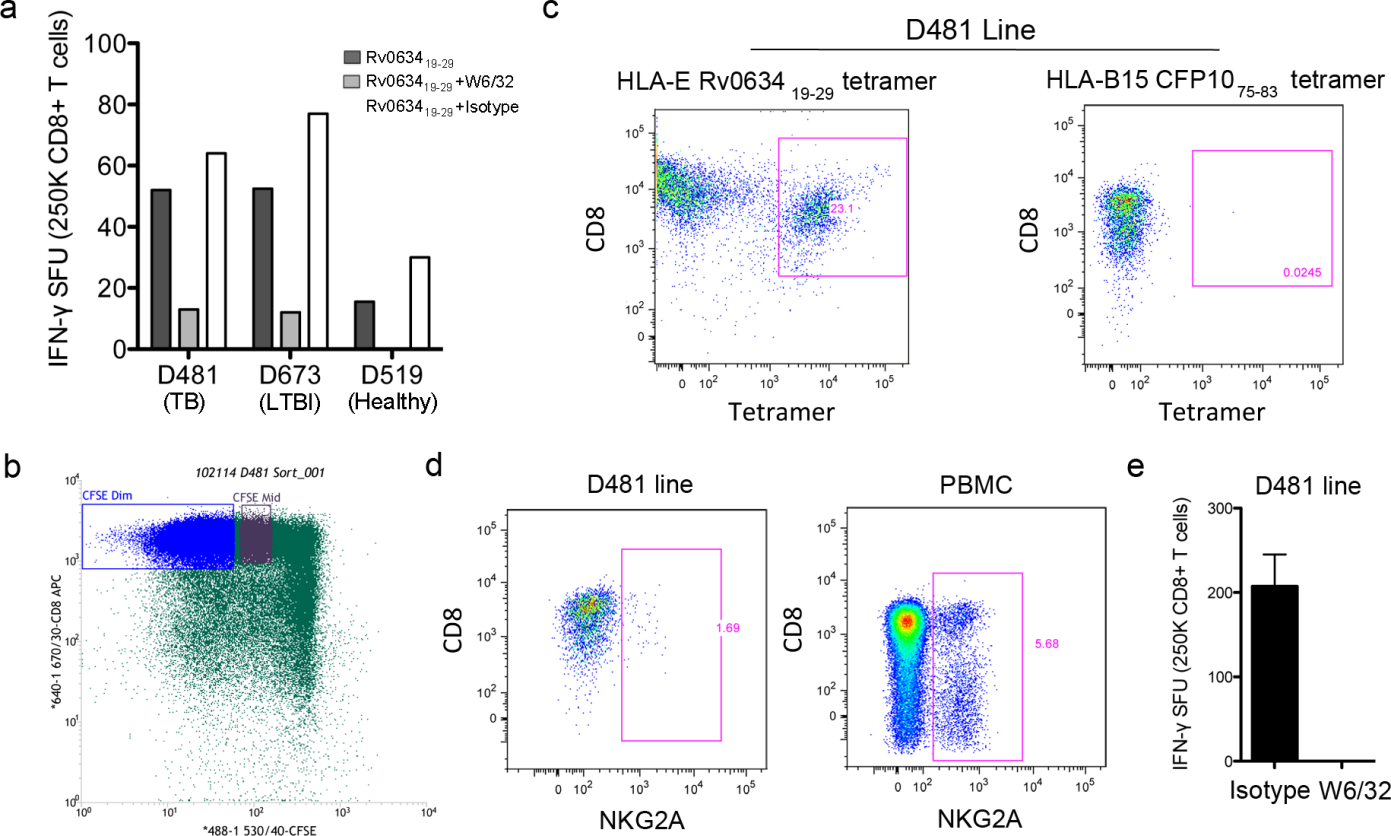
The CD8^+^ T cell response to Rv0634A_19-29_ is restricted by HLA-E. A) A549 cells were pulsed with the Rv0634_19-29_ peptide and used as antigen presenting cells in an ELISPOT assay with IFN-γ production by CD8^+^ T cells from mismatched donors as a readout. B-D) D481 CFSE-labeled CD8+ T cells were isolated and cultured with Rv0634A_19-29_ peptide-pulsed autologous macrophages and DC. CFSE-dim cells were sorted and stained with the Rv0634A_19-29_ HLA-E tetramer and antibodies against CD3 and CD8. As a control, the D481 line was also stained with a non-HLA-E tetramer (C), or with anti-NKG2A (D) do demonstrate that the tetramer staining is specific. Whole PBMC were stained with the anti-NKG2A antibody as a positive control. E) The mismatched A549 cell line was pulsed with the Rv0634A_19-29_ peptide and used as antigen presenting cells with D481 CD8^+^ T cell line.

The Rv0634A_19-29_ peptide was the most commonly recognized peptide, with CD8^+^ T cells from 13 of 16 donors responding. For 10 of those 13 donors, the response to Rv0634A_19-29_ was also the strongest, with an average of 46.5(+/-39.9) IFN-γ /SFU per 2.5e5 CD8^+^ T cells across all donors. Because this peptide had both the most common and strong response, we chose to analyze it further to validate that T cell responses to the peptide were HLA-E restricted. Given the HLA-A2 motif of many of the peptides, classically restricted CD8^+^ T cell responses to peptide-pulsed autologous DC could not be ruled out for the two HLA-A2 donors (D454 and D603). As such, we analyzed the *ex vivo* responses of CD8^+^ T cells from representative donors that could be tested with an HLA-mismatched APC. CD8^+^ T cells isolated from donor PBMC were added to Rv0634A_19-29_ peptide-pulsed A549 cells, a line that is mismatched with the selected donors at the HLA-A, B, and C alleles. Responses by CD8^+^ T cells from representative HLA-mismatched TB (D481), LTBI (D673), and healthy (D519) donors are shown in Fig 6A. The addition of the pan-HLA I specific antibody W6/32, but not an isotype control, resulted in a reduction in IFNγ production by CD8^+^ T cells from each of the donors, further supporting the HLA-E restriction of these responses to the Rv0634A_19-29_ peptide, although not ruling out HLA-F or–G restricted responses.

To confirm that the response to Rv0634A_19-29_ was HLA-E restricted, and to demonstrate that Rv0634A_19-29_ responsive, HLA-E restricted CD8^+^ T cells were present in donor PBMC, we generated tetrameric HLA-E with the Rv0634A_19-29_ peptide. The Rv0634A_19-29_ peptide was used to generate a T cell line from the CD8^+^ T cells of one of the Mtb-infected donors, D481. CFSE-diluted CD8^+^ T cells generated after culture with peptide-pulsed autologous macrophages and DC were sorted and stained with the Rv0634A_19-29_ HLA-E tetramer (Fig 6B and 6C). 23.1% of expanded CD8^+^ T cells were tetramer positive (Fig 6C). The line did not stain with a control tetramer or with an antibody against NKG2A, demonstrating peptide-specific staining by the HLA-E tetramer (Fig 6C and 6D). In an IFN-γ ELISPOT assay using peptide-pulsed HLA-mismatched DC, we observed a response by the CD8^+^ T cell line, which was blockable by W6/32 (Fig 6E). Together, these data confirm the HLA-E restriction for this epitope and rule out binding of the epitope to HLA-A2 or other Class I molecules.

## Discussion

While ongoing efforts to diagnose and treat TB disease have had a profound impact, eradication of TB disease will likely require the development of new vaccine that can either prevent infection or prevent disease progression following infection [1]. In this regard, there has been increased emphasis on the development of innovative vaccine strategies [35]. One such strategy is the targeting of T cell responses not restricted by donor HLA. We and others have demonstrated non-classical HLA-E-restricted T cell responses to Mtb-infected cells directly *ex vivo* [8, 19–21], however specific ligands remain largely uncharacterized. In this study we sought to identify pathogen-derived ligands that are processed and presented on HLA-E in the context of intracellular Mtb infection. Using a high-yield system designed to purify ligands associated with Class I molecules in the context of infection, we collected ligand-bound sHLA-E molecules from Mtb-infected cells for analysis of ligand binding by mass spectrometry. Twenty-eight novel HLA-E ligands were identified from 13 Mtb source proteins. One of these ligands, derived from the conserved hypothetical protein Rv0634A, was recognized by 13 of 16 donors tested, including healthy controls, LTBI and TB patient donors, suggesting it may have broad scope for vaccine development.

Currently, HLA-E binding peptides and binding motifs are largely defined by the binding of HLA leader sequences. Our study demonstrates that HLA-E also presents non-host peptides in the context of infection, validating the findings of Hansen et al. (2016) and Joosten et al. (2010) [15, 16]. Interestingly, although HLA-E is highly conserved, our data are consistent with other findings demonstrating that HLA-E-restricted T cells are able to recognize bacterial HLA-E epitopes that are diverse in terms of length, amino acid sequence, and modifications [15, 32]. Although the leader peptide sequences all ranged from 8–11 amino acids, Mtb peptides identified ranged in length from 8–20 amino acids with modification including oxidation and acetylation, suggesting plasticity in the HLA-E binding groove.

Although we identified 28 Mtb peptides bound to HLA-E in the context of infection, these peptides were a very small proportion (0.4%) of the ligand pool. The small contribution of Mtb ligands to the total ligand pool may be a reflection of the intracellular localization of Mtb. Following infection, viral proteins are translated in high quantities by host cell machinery in the cytosol, where they are readily available to be processed and presented through the same peptide loading complex as host cell peptides [36]. Mtb, in contrast, resides in a membrane-bound phagosome, and bacterial antigens must gain access through the membrane to the cytosol, or be processed and presented in endocytic pathways [37]. The source proteins identified for the Mtb ligands are consistent with these processing and presentation pathways. Eight of the proteins are either secreted antigens or membrane-bound proteins, which may be more likely to reach the cytosol, or be more accessible to endosomal Class I loading pathways. Of note, half of the HLA-E peptides were derived from Esx family proteins including EsxA (ESAT-6), an important secreted antigen. The ESX-1 secretion system could play a critical role in Mtb HLA-E ligands accessing the cytosol or phagosomal HLA-E. The loss of this secretion system and the associated antigens in *M*. *bovis* BCG may limit the presentation of mycobacterial ligands on HLA-E, thus reducing the expansion of HLA-E restricted T cells in the context of BCG vaccination.

Here, we used IFN-γ as a readout for T cell activity in response to peptide stimulation. Using this readout, 12 of the 28 Mtb-derived HLA-E binding peptides elicited an IFN-γ response, suggesting that T cells recognizing these peptides have Th1 like functions. This leaves the question as to why the other 16 Mtb HLA-E binding peptides did not elicit any IFNγ release by CD8^+^ T cells. One simple explanation is that we have not screened enough donors to find responses to all of the identified peptides. Another possibility is that T cells recognizing the remaining peptides have different effector functions. Interestingly, recent studies that followed up on the Mtb HLA-E binding peptides predicted through bioinformatics analysis [16] demonstrated that certain HLA-E restricted Mtb reactive CD8^+^ T cell clones have unconventional phenotypes that more closely resemble Th2 type functions [17, 18]. These results emphasize the importance of considering that non-classical T cells, such as those restricted by HLA-E, may have non-traditional effector functions. Furthermore, these findings underscore the importance of using these diverse and complementary approaches to identifying epitopes for donor-unrestricted T cells.

HLA-E is an attractive candidate for novel and innovative vaccine strategies for TB. As previously mentioned, it is highly monomorphic, is not down-regulated after HIV infection, and has recently been shown to play an important role in generating SIV-reactive T cells following vaccination. We have identified several Mtb epitopes that are presented by HLA-E in the context of intracellular Mtb infection. Further analysis of the T cells elicited through recognition of these peptides will increase our understanding of the HLA-E-restricted cellular response to infection with Mtb.
